# Wunderlich Syndrome: Spontaneous Cystic Rupture on Account of Acquired Kidney Atrophy

**DOI:** 10.7759/cureus.30386

**Published:** 2022-10-17

**Authors:** Theodoros Mariolis-Sapsakos, Eirini Nannou, Stavros Angelis, Dimitrios Filippou

**Affiliations:** 1 Surgical Anatomy, National and Kapodistrian University of Athens Medical School, Athens, GRC; 2 Radiology, General Oncology Hospital of Kifisia “Agioi Anargyroi”, Athens, GRC

**Keywords:** cystic rupture, kidney hypoplasia, lenk’s triad, kidney atrophy, wunderlich syndrome

## Abstract

Wunderlich syndrome is an uncommon condition of spontaneous subcapsular and perirenal hemorrhage of atraumatic etiology in the kidney, with the potential to spread to the retroperitoneal region beyond the perirenal fascias. Its clinical manifestations usually include Lenk’s triad, namely, acute flank pain, flank mass, and hemodynamic instability, which vary depending on the causative underlying renal pathology. Tumor bleeding of benign and malignant renal neoplasms is the most common cause of this syndrome, followed by vascular disorders and renal cystic diseases. Here, we report the case of a unilateral subcapsular renal hematoma on account of a left atrophic kidney with parapelvic cystic formations and variant hypoplastic vasculature which was successfully managed via radical nephrectomy after initial conservative treatment. Spontaneous cystic rupture contributed to the emergence of the syndrome, and its mechanisms are being addressed.

## Introduction

Wunderlich syndrome constitutes an uncommon condition of spontaneous subcapsular and perirenal hemorrhage of atraumatic etiology in the kidney, with the potential to spread to the retroperitoneal region beyond the perirenal fascias (hemoretroperitoneum). Its clinical manifestations usually include Lenk’s triad, namely, acute flank pain, flank mass, and hemodynamic instability, which can easily be misdiagnosed with other acute abdomen conditions, such as urolithiasis, acute appendicitis, and bowel perforation. Fever, macroscopic or microscopic hematuria, systemic arterial hypertension, nausea, and vomiting might also be present. Angiomyolipoma is the most common benign and renal cell carcinoma is the most common malignant neoplastic cause. Less common causes include problematic vasculature such as polyarteritis nodosa, rupture of renal artery aneurysms, segmental arterial mediolysis, arteriovenous malformations or fistulas, vasculitis, and cystic medial necrosis. Rupture of renal cysts such as in polycystic kidney diseases, infections, nephrolithiasis, and coagulopathies have also been reported as etiological factors. Contrast-enhanced computed tomography (CT) is the gold standard for an initial diagnosis [1,2]. The optimal treatment modality is determined by the underlying pathology and the subsequent hemodynamic state. Conservative management with fluid resuscitation, analgesics, and antibiotics is opted for uncomplicated stable hemorrhagic states, whereas exploratory laparotomy and partial or radical nephrectomy are suitable in more aggressive cases. Minimally invasive procedures such as selective renal artery embolization, radiofrequency ablation, cryoablation, or microwaves are used in vascular diseases and renal tumors [3-5].

## Case presentation

**Figure 1:**
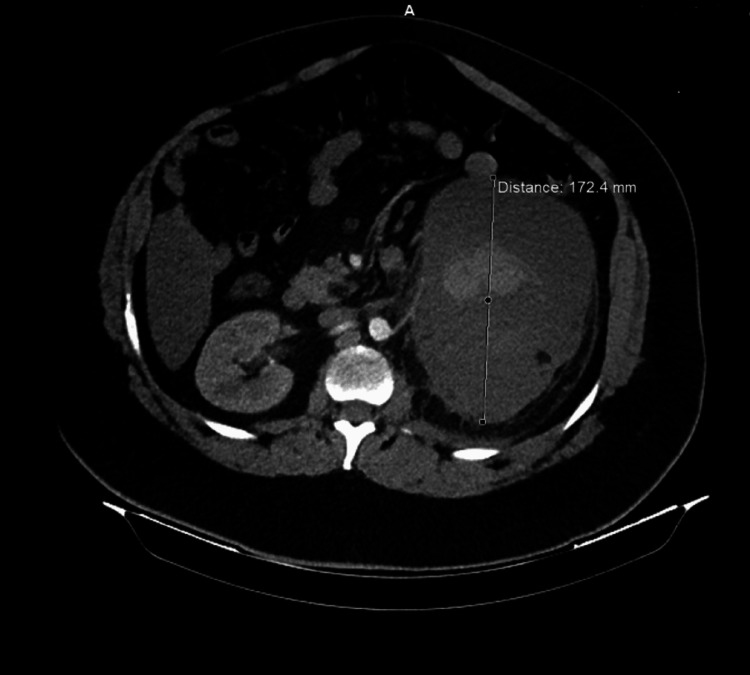
Hematoma with a diameter of 17 cm confined within the perirenal fascias on contrast-enhanced computed tomography scan.

A 31-year-old Caucasian male, with a history of asthma and obesity, reported a first episode of colic-like pain in the left flank area in his 20s. CT scan, at that time, revealed an atrophic left kidney with parapelvic cysts that were left unsupervised, and no calculi disease was observed. Ten years later, he experienced a sudden continuous severe pain in the left flank that lasted for approximately 10 hours until he sought help at our emergency department. Upon admission to our institution, he presented with tachycardia, cold intolerance, anemia, and a distended left flank. Hematuria was not observed, and the pain had moderately subsided. He underwent a contrast-enhanced CT scan that revealed a 17 cm maximum diameter hematoma in the left retroperitoneal region confined within the perirenal fascias that displaced the adjacent intestines (Figure 1). The patient remained hospitalized for a few days to further evaluate the hemorrhage. The hematoma was localized and hemodynamic stability was restored; therefore, he was discharged and given instructions to avoid physical exertion, anticoagulant use, and keep track of his hematocrit and serum creatinine levels regularly until surgery was performed. Upon discharge from the hospital, he underwent renal scintigraphy at a private healthcare unit that demonstrated complete renal impairment.

**Figure 2:**
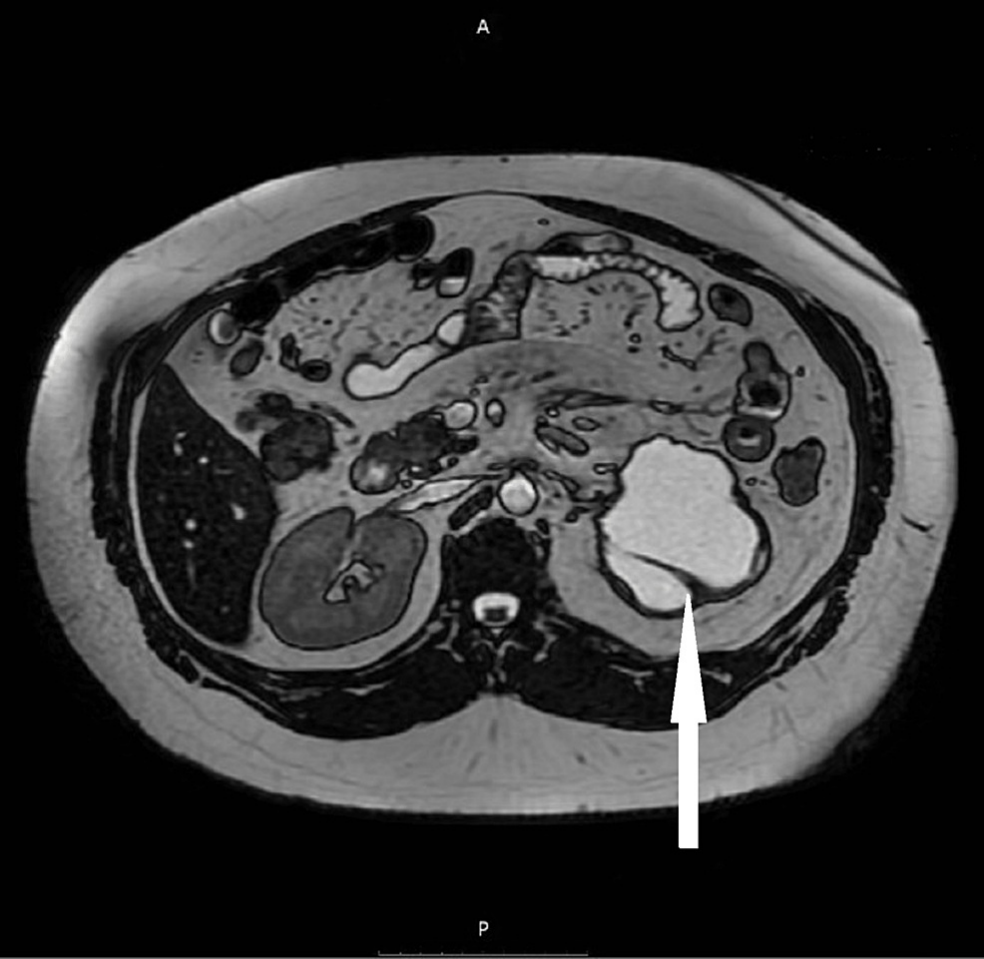
Cross-section magnetic resonance angiography of the abdomen indicating shrinkage of the hematoma and intracystic hemorrhage.

**Figure 3:**
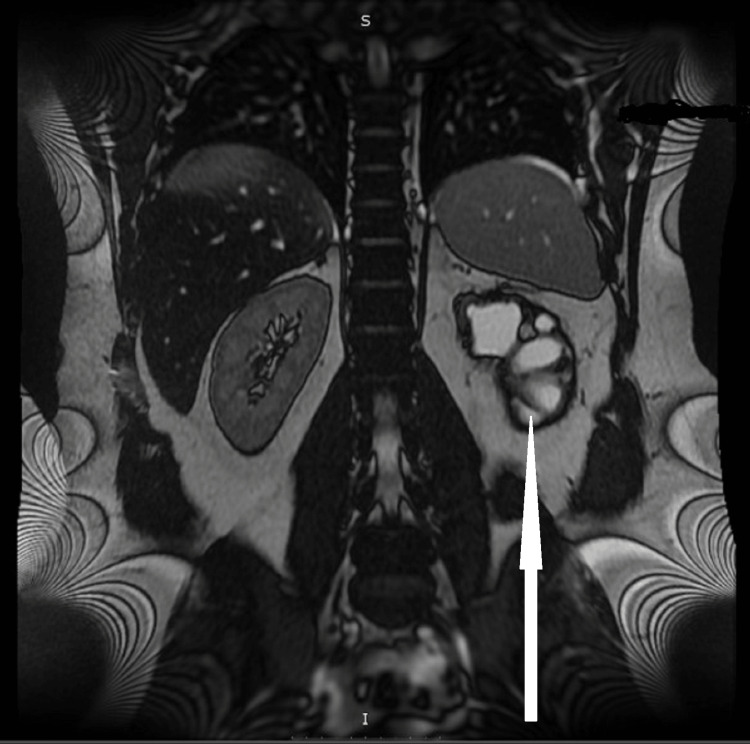
Coronal section magnetic resonance angiography of the abdomen indicating shrinkage of the hematoma and intracystic hemorrhage.

**Figure 4:**
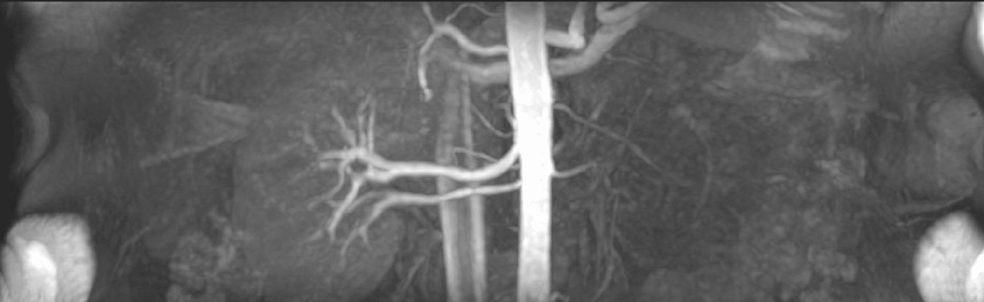
Magnetic resonance angiography indicating double renal artery in the right kidney and variant vasculature in the left kidney.

Three months later, magnetic resonance (MR) angiography of the abdomen showed a significant decrease in the size of the hematoma and revealed multiple hypoplastic vessels of the kidney hilum, cystic degeneration, shrinkage of the renal parenchyma, and thickening of the surrounding fascia (Figures 2, 3). The initial hemorrhage seemed to originate from inside the cystic formations. The right kidney showed a double renal artery (Figure 4).

**Figure 5:**
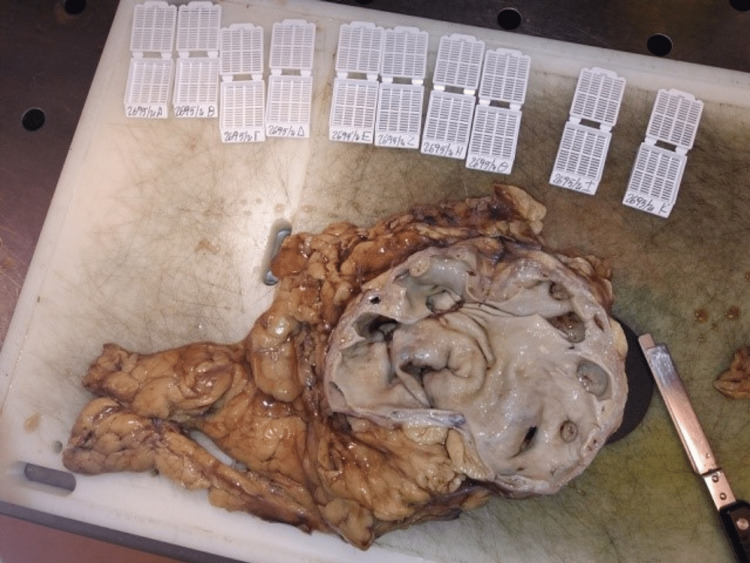
Pelvicalyceal dilatation.

**Figure 6:**
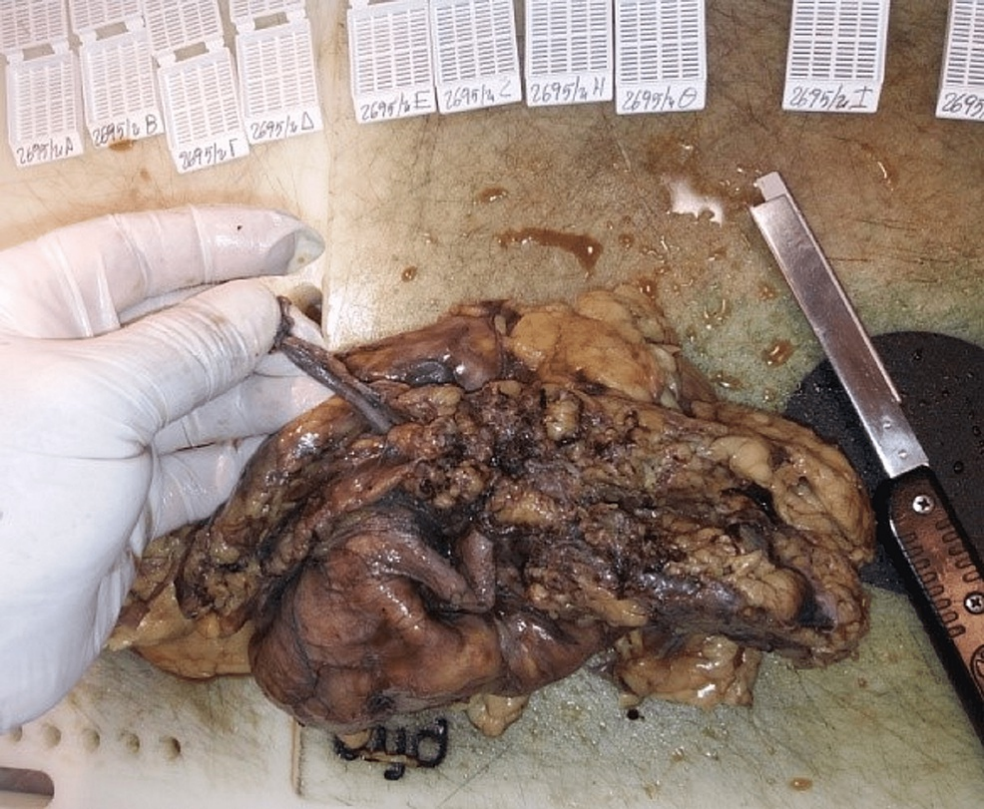
Renal artery variant.

Subsequently, surgery was scheduled and an open midline incision radical nephrectomy was performed (Figures 5, 6). It included the removal of the left kidney, the left para-aortic lymph nodes, part of the left ureter (6.5 cm length), the perirenal fat that contained the adrenal gland at the level of the upper renal lobe, five lymph nodes at the hilar level, and part of the greater omentum. The operation was uneventfully completed and the patient had a quick recovery.

The surgical pathology report documented pelvicalyceal dilatation, multiple parapelvic cystic formations of varying sizes with a smooth inner surface of flattened simple cuboid epithelium containing clear serous liquid, complete disfiguration of the normal renal parenchyma morphology, diffuse hemosiderin infiltrates, interstitial fibrosis, a few sclerotic glomeruli, tubular atrophy, and a few chronic inflammatory infiltrates of the parenchyma. The hilar vessel wall was dilated and thickened, with elements of acute inflammation, whereas in the hilar fat, acute and chronic inflammatory infiltrates were found. Adrenal hyperplasia was also observed.

## Discussion

In the present case, no apparent single causal link could be easily tracked between the underlying kidney pathology and the sudden hemorrhage. Taking into consideration the fact that we have insufficient data about the state of the kidney before the hemorrhage, we can only presume the etiology retrospectively as multifactorial. Kidney atrophy was reported 10 years ago and the MR angiography reported more than three hypoplastic arteries. The patient never exhibited arterial hypertension. In addition, there was localized cystic renal disease mainly in the pelvicalyceal system [6,7]. No aneurysms or neoplastic lesions were detected from the pathology report. The patient reported no trauma, and the clinical examination was negative.

Kidney atrophy is an acquired condition of diminution in kidney size usually caused by depletion in blood supply (e.g., renal artery stenosis), inflammatory states such as pyelonephritis, calculi disease causing obstructive hydronephrosis, malnutrition, and hormonal imbalance [8-10]. Kidney hypoplasia is a congenital condition that mostly manifests in childhood. There are three types of hypoplastic states that involve deficits in the ureteric bud formation and present with fewer renal lobes than normal: segmental, oligomeganephronia, and simple hypoplasia. There is also cortical hypoplasia and oligonephronia where fewer nephrons are produced. In oligonephronia, there is a high risk for hypertensive states and kidney failure in adult life [11,12]. In our case, an acquired morphology was observed probably due to decreased blood supply in the organ as the hilar vessels were found numerous yet hypoplastic [13,14].

According to the literature, kidney cystic rupture is a rare phenomenon. The sustainability of the cyst is preserved by equilibrium among intracystic and extracystic pressures and the stability of the cystic wall [15,16]. An increase in intracystic pressures can be either idiopathic, where increased production of cystic fluid causes quick cyst enlargement that ultimately leads to wall atrophy and rupture, or caused by intracystic hemorrhage usually presenting as hypertension, coagulopathies, or seen in kidney infections. Communication between a bleeding cyst and the adjacent collecting system usually leads to hematuria [17]. In our case, hematuria was not observed. The cystic wall can be torn in trauma, become atrophic in sudden elevation of intracystic pressures, or become frail during an infection. Thus, the blood vessels surrounding it are torn down as well and hemorrhage occurs. An increase in extracystic pressures can occur due to hemorrhage (hypertension, ruptured aneurysms, bleeding tumor, etc.), trauma (intrarenal hemorrhage, blunt trauma), due to an increase in intrapelvic pressure as in ureterolithiasis, prostate enlargement, neurogenic bladder, neoplasms compressing the urinary tract, or, as in our case, kidney atrophy. The atrophic kidney is fibrotic with diminished expansion capacity and elasticity, compressing the expanding cyst externally [18-20].

The preferred therapeutic approach was determined by the hemodynamic state of the patient and the extent of the damage. There was no need for embolization or immediate exploratory laparotomy as there were no signs of active bleeding or hemodynamic deterioration. Furthermore, the kidney was completely impaired, as shown on the scintigraphy examination. The surgeon proceeded to radical nephrectomy upon the recession of the hematoma, thus preventing further complications arising from a necrotic organ.

## Conclusions

We can presume that spontaneous cystic rupture is the prominent cause of Wunderlich syndrome as a complication of kidney atrophy induced by chronic hypoperfusion. Emergency physicians need to be aware of this entity as it masquerades other acute abdomen conditions. Moreover, the need for a regular check-up on alterations in the size of renal cysts is highlighted as it might prevent a hemorrhagic rupture with possibly lethal consequences.
